# Abstracts from the 72^nd^ Annual Meeting of the British Thyroid Association

**DOI:** 10.1186/s13044-024-00203-w

**Published:** 2024-06-14

## L1 George Murray lecture: Personalizing thyroid epidemiology

### Graham Leese

#### Ninewells hospital and Medical School, Dundee, UK

##### **Correspondence:** Graham Leese (graham.leese2@nhs.scot)

*Thyroid Research 2024*, **17(S1):**L1

We tend to think of genetics when considering personalized medicine, but all aspects of a medical history, examination and tests are used to “personalize” care. Large epidemiological studies demonstrate that thyroid disease is associated with cardiovascular events, especially dysrhythmias, and osteoporosis as the most significant co-morbidities. Low and High serum TSH concentration are markers of risk for people on thyroid replacement. We have shown that the lowest risk is associated with a serum TSH of about 0.4-1.0mU/l. Epidemiological studies suggest the need for age related reference ranges and provide data on the risks of untreated subclinical hyperthyroidism and hypothyroidism and use of long-term liothyronine prescribing. Genetics have transformed personalized care into ‘precision care’. Thyroid cancer exemplifies the use of specific drugs to treat tumours with specific mutations such as BRAF. However the role of genetics in routine thyroid care is less developed. Identification of specific DIO2 polymorphisms has been controversial in the use of liothyronine treatment. Our genetic epidemiology database has replicated key SNPs associated with serum TSH estimates and diagnosis of hypothyroidism. A genetic risk score (GRS) using multiple SNPs associated with TSH concentration explained nearly 12% of the variation in serum TSH. The GRS also predicted fracture in men. In a preliminary mendelian randomisation study, atrial fibrillation was associated with the INSR ‘GG’ genotype in people on thyroxine. Linking in with a European thyroid genetic collaboration, named ThyroidOmics, has allowed further developments. The future should focus on combining both clinical and genetic criteria to predict individual risk.

## S1 Updated classification of thyroid pathology: what the clinician needs to know

### Fernando Schmitt

#### Medical Faculty of Porto University, RISE (Health Research Network), Porto, Portugal

##### **Correspondence:** Fernando Schmitt (fschmitt@med.up.pt)

*Thyroid Research 2024*, **17(S1):**S1

The 5th edition of the WHO Classification of Endocrine and Neuroendocrine Tumors introduces significant updates concerning thyroid gland tumors. This new edition refines the categorization of thyroid tumors, enhancing the understanding of their origins, pathological features, molecular characteristics, and biological behaviors. The key updates in the classification are:

New Categories of Thyroid Tumors

- Benign Tumors: The category includes traditional follicular adenomas and introduces variants with diagnostic and clinical relevance, such as those with papillary architecture and oncocytic adenomas.
- Low-Risk Neoplasms: This includes non-invasive follicular thyroid neoplasm with papillary-like nuclear features (NIFTP), thyroid tumors of uncertain malignant potential, and hyalinizing trabecular tumor.
- Malignant Neoplasms: These are now stratified more distinctly by molecular profiles and aggressiveness. Papillary thyroid carcinomas (PTCs) with various morphological subtypes are categorized under BRAF-like malignancies, while RAS-like malignancies include invasive encapsulated follicular variant PTC and follicular thyroid carcinoma.

Revision of Existing Classifications

- The criteria for the tall cell subtype of PTC have been revised.
- Cribriform-morular thyroid carcinoma is reclassified and no longer considered a subtype of PTC.
- The term “Hürthle cell” is discouraged, with a shift towards recognizing oncocytic carcinoma as a distinct entity.
- Anaplastic thyroid carcinoma remains the most aggressive form. Squamous cell carcinoma is now considered a subtype of anaplastic carcinoma.

High-Grade Follicular Cell-Derived Malignancies

- This new category encompasses both poorly differentiated carcinoma and high-grade differentiated thyroid carcinomas, noted for similar clinical behaviors due to increased mitotic activity and necrosis.

Medullary Thyroid Carcinoma

- Medullary carcinomas retain their unique classification and are now graded based on mitotic count, tumor necrosis, and Ki67 labeling index.

New Sections for Unusual Neoplasms

- Salivary gland–type carcinomas like mucoepidermoid carcinoma and secretory carcinoma now have their dedicated section.
- Thymic tumors within the thyroid and a rare thyroblastoma associated with DICER1 mutations are newly included categories.

Emphasis on Biomarkers

- The revised classification highlights the importance of biomarkers in diagnosing and prognosticating thyroid tumors, supporting more tailored therapeutic strategies.

This revised classification aims to provide a more nuanced understanding of thyroid tumors, facilitating better diagnosis, management, and research into these diverse conditions. This can significantly impact clinical practice and patient outcomes by allowing for more precise classification and targeted treatment approaches.

## S2 Molecular profiling for the diagnosis and management of advanced thyroid cancer

### Jon Wadsley

#### Weston Park Cancer Centre, Sheffield Teaching Hospital NHS Foundation Trust, Sheffield, UK

##### **Correspondence:** Jon Wadsley (jonathan.wadsley1@nhs.net)

*Thyroid Research 2024*, **17(S1):**S2

The oncological treatment of many cancers has been transformed in recent years by the development of drugs targeted at specific molecular genetic alterations. When applied to patients with cancers harbouring the relevant mutation these drugs can control advanced disease for significant lengths of time, often with less toxicity than with conventional therapies.

These developments are now impacting the management of advanced thyroid cancers, where it is reported that as many as 40% of cases may carry targetable genetic alterations. In this session we will consider the types of genetic alterations that can be found in advanced thyroid cancer (medullary thyroid cancer, iodine refractory differentiated thyroid cancer and anaplastic thyroid cancer), both germline and somatic. We will review the emerging evidence for the benefits of drugs targeting specific alterations including RET alterations, NTRK fusions and BRAF mutations in advanced thyroid cancers, and which of these drugs are currently available in UK practice.

We will also consider the practical aspects of how and when to arrange molecular genetic testing in the UK setting to ensure that patients with advanced thyroid cancer are able to access these novel therapies.

## S3 Genetics of syndromic thyroid cancers

### Hannah Nieto

#### University of Birmingham, Birmingham, UK

##### **Correspondence:** Hannah Nieto (h.nieto@bham.ac.uk)

*Thyroid Research 2024*, **17(S1):**S3

Twenty-five percent of medullary thyroid cancers are inherited and for differentiated thyroid cancer 5-15% are due to germline mutations. Both sporadic and germline medullary thyroid cancers are strongly linked to mutations in the RET gene, including MEN2A & MEN2B syndrome. Different RET mutations confer different cancer risks and are managed proportionally. Co-ordinating management of adrenal and other extra thyroidal manifestations within a multidisciplinary team is a key part of patient care. Differentiated thyroid cancer has a wide range of potential causative mutations, with inherited syndromes that confer an increased thyroid cancer risk including Cowden syndrome, familial adenomatous polyposis, DICER1 syndrome and Li-Fraumeni syndrome, amongst others. These syndromes also have extra thyroidal aspects to their care, often including age linked screening, lending a complexity to their care. Several syndromes are linked to critical thyroid genes that are also implicated in sporadic (somatic) thyroid cancers. This talk will consider the most common syndromic thyroid cancers, and cover their clinical care as well as the molecular genomics behind the syndromes.

## S4 Year in basic thyroidology

### Graham R Williams

#### Imperial College London, London, UK

##### **Correspondence:** Graham R Williams (graham.williams@imperial.ac.uk)

*Thyroid Research 2024*, **17(S1):**S4

In this presentation I will discuss the best basic science papers in the thyroid field published during the last year. All basic science papers in the fields of thyroid gland development and function, thyroid autoimmunity, thyroid cancer, and thyroid hormone action published between June 1st 2023 and May 31st 2024 were evaluated. Searches of PubMed (https://pubmed.ncbi.nlm.nih.gov) and bioRxiv (https://www.biorxiv.org) identified over 2000 articles. Initially, about 100 of those considered to be the most impactful were selected based on their novelty, scientific rigor, general interest, and application of cross disciplinary, state-of-the-art methods. Forty-five papers were selected for in-depth scrutiny. Ultimately, 5 papers will be presented in depth and a further 10 discussed to provide a comprehensive overview of the current state-of-the-art in basic thyroidology.

## S5 Immunotherapy for thyroid eye disease

### Mario Salvi

#### Graves’ Orbitopathy Center, Fondazione IRCCS Cà Granda, Milan, Italy

##### **Correspondence:** Mario Salvi (mario@mariosalvinet.it)

*Thyroid Research 2024*, **17(S1):**S5

Oral and intravenous glucocorticoid (GC) have been used for decades off label in active moderate-severe TED and have shown to be effective in inactivating disease although. Randomized studies comparing IVGC vs OGC have reported an 80% response rate with IVGC versus 50-60% with OGC, generally with dosing not exceeding 8 g, recommended as a total cumulative dose to reduce potential side effects. Non-steroidal oral immunosuppressive agents as mycophenolate mofetil, cyclosporine A and azathioprine offer modest benefit in TED. Of the immunomodulatory monoclonal antibodies, only rituximab and tocilizumab have been studied in randomized controlled trials and have been shown to be effective in inactivating TED and also in affecting disease relapse rate. Recently, teprotumumab, an insulin-like growth factor-1 receptor (IGF-1R) inhibitor, has been approved by FDA for the treatment of TED. Teprotumumab in two randomized trials has demonstrated significant improvement in proptosis, clinical activity score, diplopia, and quality of life in patients with active TED. This drug has been studied also in patients with chronic TED in a recent trial. Evidence of adverse auditory complications are under further investigation. Newly proposed TED therapies, currently in pre-clinical and clinical trial phases, include thyroid stimulating hormone (TSH) receptor inhibitory drugs, RVT-1401, satralizumab, IGF-1R drugs delivered subcutaneously and orally.

## S6 Immunotherapy-related thyroid dysfunction

### Safwaan Adam

#### University of Manchester, Manchester, UK

##### **Correspondence:** Safwaan Adam (safwaan.adam1@nhs.net)

*Thyroid Research 2024*, **17(S1):**S6

Immune checkpoint inhibitors have revolutionised cancer therapy and are now being increasingly used across different primary cancer disease sites. However, due to the immune system activation, inflammatory side effects are common and can cause significant morbidity. Endocrine organs are particularly susceptible to the effects of immune checkpoint inhibitors and represent amongst commonest side-effects. The thyroid gland is especially prone to immune checkpoint inhibitor induced inflammation and the impact is usually seen in the form of an inflammatory thyroiditis which frequently results in irreversible permanent hypothyroidism after a transient thyrotoxic phase. In this session, the prevalence and natural history immune checkpoint mediated thyroid function abnormalities are discussed using evidence from randomised clinical trial data as well as real-world data from large cohort studies. There will also be illustration of the pattern of inflammatory insults using case studies.

## OR1 Single-cell analysis for the human developing thyroid uncovers thyrocyte heterogeneity and active interactions during development

### Hassan Massalha^1,2^, Mi Trinh^1^, Iva Kelava^1^, Cecilia Icoresi-Mazzeo^1^, Nadia Schoenmakers^3^, Sam Behjati^1^, Roser Vento-Tormo^1^

#### ^1^Wellcome Sanger Institute, Cambridge, UK; ^2^Theory of Condensed Matter Group, Cavendish Laboratory, University of Cambridge, Cambridge, UK; ^3^University of Cambridge Metabolic Research Laboratories, Wellcome Trust-Medical Research Council Institute of Metabolic Science, Addenbrooke’s Hospital, Cambridge, UK

##### **Correspondence:** Hassan Massalha (hm11@sanger.ac.uk)

*Thyroid Research 2024*, **17(S1):**OR1

**Background:** Normal functioning of the thyroid is of profound importance for lifetime health due to its role in hormone production. Dysfunction of the thyroid is associated with severe congenital pathologies, some of them appearing in childhood. For example, over half the babies born with congenital hypothyroidism appear completely normal and without symptoms. However, early diagnosis of thyroid defects is lacking mainly due to a poor understanding of the development of the tissue in utero.

**Aims:** Decipher human thyroid tissue development dynamics and cellular, and their correlation with thyroid function.

**Methods:** To address the above aims we using single cell genomics and spatial transcriptomics. To validate our findings, we employed advanced fluorescent microscopy and multiplexed RNAscope imaging techniques.

**Results:** Here we have established a comprehensive spatiotemporal atlas of the developing human thyroid during the first and second trimester of pregnancy. Our dense profiling of more than 250k cells using single-cell sequencing has revealed the main cell types, their developmental relationships and transcription factors leading to the formation of the thyroid gland. We characterised the early development thyroid specific cell types including thyrocytes, parathyroid gland and parafollicular cells, known as C-cells. Notably, we found that thyrocytes are heterogeneous epithelial populations and split thyroid-hormones production between different subsets. We further validated the spatial heterogeneity of thyrocyte subpopulations using multiple spatial transcriptomics methods. Lastly, we derived ligand-receptor interactions that drive the maturation of thyrocytes during development. Our results confirm the division of labour of the thyrocytes, and highlight active cell-cell communications during thyroid gland development.

**Conclusions:** Altogether our analysis exemplifies the division of labour principle observed in other adult tissues also applies to the development of the thyroids, expanding our knowledge of thyroid-hormones synthesis and regulation. Future work includes how the function principles and potential interactions are altered in pathological conditions.

## OR2 Transcriptional reprogramming using a new dual NIS agonist enhances radioiodide uptake with implications for predicting thyroid cancer recurrence

### Katie Brookes^1^, Ling Zha^1^, Jana Kim^2^, Sarinya Wongsanit^1^, Jessica Fear^1^, Benjamin Small^3,4^, Hannah R. Nieto^1^, Vinodh Kannappan^3,4^, Weiguang Wang^3,4^, Sissy Jhiang^5^, Matthew D. Ringel^5^, Moray J. Campbell^6^, Kavitha Sunassee^2^, Philip J. Blower^2^, Kristien Boelaert^7^, Vicki E. Smith^1^, Martin L. Read^1^, Christopher J. McCabe^1^

#### ^1^Institute of Metabolism and Systems Science (IMSS), and Centre of Endocrinology, Diabetes and Metabolism (CEDAM), University of Birmingham, Birmingham, UK; ^2^School of Biomedical Engineering & Imaging Sciences, King’s College London, London, UK; ^3^Research Institute in Healthcare Science, Faculty of Science and Engineering, University of Wolverhampton, Wolverhampton, UK; ^4^Disulfican Ltd, University of Wolverhampton Science Park, Wolverhampton, UK; ^5^Division of Endocrinology, Diabetes, and Metabolism and Cancer Biology Program, The Ohio State University College of Medicine and Comprehensive Cancer Center, Columbus, Ohio, USA; ^6^Division of Cancer Biology, Cedars Sinai Cancer, Los Angeles, USA; ^7^Institute of Applied Health Research, University of Birmingham, Birmingham, UK

##### **Correspondence:** Katie Brookes (k.baker.2@bham.ac.uk)

*Thyroid Research 2024*, **17(S1):**OR2

**Background:** New approaches are urgently needed to enhance radioiodide (RAI) ablation of aggressive thyroid cancer. Previously, we reported that valosin-containing protein inhibitors (VCPi), such as disulfiram, markedly increase sodium iodide symporter (NIS) activity to promote RAI uptake. Disulfiram inhibits NPL4 activity – a critical VCP cofactor – via its copper bound diethyldithiocarbamate metabolite Cu(DDC)_2_. We hence hypothesised that disulfiram and its metabolites increase RAI uptake by interfering with ER-Associated Degradation (ERAD) via a VCP/NPL4 pathway, permitting more NIS protein to be trafficked to the plasma membrane.

**Aims:** To determine the mechanistic impact and clinical relevance of Cu(DDC)_2_ in RAI therapy.

**Methods:** We utilised RNA-Seq to identify Cu(DDC)_2_-regulated transcriptional pathways. NIS function was monitored in wild-type BALB/c mice via Technetium-99m pertechnetate (^99m^Tc) uptake following intravenous administration. TCGA was appraised to investigate Cu(DDC)_2_-gene interactions in recurrent RAI-treated papillary thyroid cancer (PTC).

**Results:** Cu(DDC)_2_ increased RAI uptake in multiple thyroid cancer cell lines (mean~3.4-fold). Subsequent RNA-Seq revealed potent transcriptional changes in Cu(DDC)_2_-treated 8505C cells (4661 genes; *P*<0.05). TaqMan RTPCR confirmed induction of transcription factors with key roles in regulating NIS expression, including PAX8, in thyroid cancer cell lines and human primary thyrocytes. In support, Cu(DDC)_2_ was unable to induce NIS mRNA or ^125^I uptake when PAX8 was depleted. Importantly, induction of thyroidal ^99m^Tc-uptake (~35%;*n*=6-11;3mg/kg; *P*<0.05) in BALB/c mice treated intraperitoneally with Cu(DDC)_2_ was associated with increasing PAX8 mRNA (1.4-fold; *P*<0.01). Additionally, Cu(DDC)_2_ retained activity in the absence of NPL4 but not VCP in thyroid cancer cells and primary thyrocytes. LASSO regression analysis using TCGA further identified a 22-gene riskscore classifier based on Cu(DDC)_2_-associated transcription factors, which showed a significantly worse prognosis in RAI-treated PTC [Hazard Ratio=11.6;95%CI 5.8-23.31; *P*<0.001;*n*=256].

**Conclusions:** Our work demonstrates that a new dual NIS agonist targets transcriptional and VCP pathways to enhance RAI uptake, with clinical relevance in impacting therapy and patient stratification for predicting recurrence.

## OR3 Results of the UK Anti-Thyroid Drug (ATD) study

### Lydia Grixti^1^, Rachel E Holliday^1^, Kath Allinson^1^, Will Drake^2^, Krishna Chatterjee^3^, Amit Allahabadia^4^, Kristien Boelaert^5^, Salman Razvi^6^, Colin Dayan^7^, Florian Wernig^8^, Onyebuchi Okosieme^9^, Jackie Gilbert^10^, Ahmed Al‑Qaissi^11^, Tristan Richardson^12^, Anastasios Gazis^13^, Zin Zin Htike^13^, Moulinath Banerjee^14^, Vladmir Vaks^15^, Christine May^16^, Rupa Ahulwalia^17^, James McClaren^18^, Prakash Abraham^19^, Graham Leese^20^, Nidhi Choudhary^21^, Simon H Pearce^1^, Bijay Vaidya^22^

#### ^1^Newcastle University, Newcastle upon Tyne, UK; ^2^Barts Health NHS Trust, London, UK; ^3^Cambridge University Hospitals NHS Foundation Trust, Cambridge, UK; ^4^Sheffield Teaching Hospital NHS Foundation Trust, Sheffield, UK; ^5^Birmingham University, Birmingham, UK; ^6^Gateshead Health NHS Foundation Trust, Gateshead, UK; ^7^Cardiff University, Cardiff, UK; ^8^Hammersmith Hospital, London, UK; ^9^Cwm Taf Morgannwg University Health Board, Pontypridd, UK; ^10^King’s College Hospital, London, UK; ^11^St James’ University Hospital, Leeds, UK; ^12^University Hospitals Dorset NHS Foundation Trust, Bournemouth, UK; ^13^Nottingham University Hospital, Nottingham, UK; ^14^Bolton Hospital, Bolton, UK; ^15^Great Western Hospital, Swindon, UK; ^16^Oxford University Hospitals NHS Foundation Trust, Oxford, UK; ^17^Norfolk & Norwich University Hospitals NHS Foundation Trust, Norwich, UK; ^18^University Hospital Wishaw, Wishaw, UK; ^19^Aberdeen Royal Infirmary, Aberdeen, UK; ^20^Ninewells Hospital, Dundee, UK; ^21^Derriford Hospital, Plymouth, UK; ^22^University of Exeter, Exeter, UK

##### **Correspondence:** Lydia Grixti (lydia.grixti@newcastle.ac.uk)

*Thyroid Research 2024*, **17(S1):**OR3

**Background:** Antithyroid drugs (ATDs) remain the mainstay of treatment for hyperthyroidism. Over 716,000 ATD prescriptions were reported in England and Wales for the financial year 2022/2023. Idiosyncratic reactions with ATDs include ATD-associated agranulocytosis (ATD-Ag) that occurs in 1 in 500 patients and fulminant hepatoxicity (ATD-H). We perform an observational, multi-centre study to identify individuals at risk.

**Methods:** A national, multi-centre survey was conducted with contributions from the Society for Endocrinology (UK) thyroid network to look at case reports of ATD-Ag (neutrophils ≤0.5x10^9^/l) , ATD induced neutropenia (ATD-N) (neutrophils between 0.5-1.0 x10^9^/l ) and ATD-H (serum alanine transferase [ALT] or alkaline phosphatase [ALP] 5x the upper limit of normal [ULN], or ALT 3xULN with bilirubin 2xULN) over 2007-2017 and prospectively over the 2-year study period.

**Results:** This study recruited a total of 60 patients from 22 UK centres. The female:male ratio was 53:7. Carbimazole (CBZ) to propylthiouracil (PTU) use was 3.3:1.

For the combined cohort of ATD-Ag and ATD-N, the median age to reaction was 43.5 and 39 years for CBZ and PTU respectively with a median duration of treatment of 34.5 and 46 days respectively.

7 patients suffered ATD-H. All were female and had Graves’ Disease. The ratio of CBZ to PTU was 4:3. The median age to reaction was 47 years. The median duration of treatment was 35 days. 1 patient taking PTU required liver transplant.

**Conclusion:** Our cohort demonstrates a predominantly white, female population in their forties taking ATDs for Graves’ disease and a median time to reaction of 34-46 days. The predominant ATD was carbimazole although we recognise that PTU was over-represented in patients with drug reactions (5 times greater) compared to the reported national number of prescriptions. We demonstrate a mortality of 0% for our cohort when compared to the historical UK cohort from yellow card data that reported 10% mortality between the years 1991-2000.

## OR4 The interactions of thyroid stimulating monoclonal autoantibody M22^TM^ with the TSHR – analysis by cryo-EM

### R. Nunez Miguel, P. Sanders, L. Allen, M. Evans, M. Holly, W. Johnson, A. Sullivan, J. Sanders, B. Rees Smith

#### FIRS Laboratories, RSR Ltd, Parc Ty Glas, Llanishen, Cardiff, UK

##### **Correspondence:** R. Nunez Miguel (firs@rsrltd.eclipse.co.uk)

*Thyroid Research 2024*, **17(S1):**OR4

**Background and Aims:** The cryo-EM structures of the TSHR in complex with the blocking autoantibody K1-70^TM^ and the TSH superagonist TR1402 have been determined. Both ligands interact only with the TSHR extracellular domain (ECD; residues 22-414). We now describe the cryo-EM structure of the stimulating autoantibody M22^TM^ in complex with the TSHR-ECD and a comparison of its interactions with the receptor with those of K1-70^TM^ and TR1402.

**Methods:** Full length human TSHR combined with M22^TM^ Fab was solubilised, purified to homogeneity and applied to cryo-EM grids. Data collection was performed on a Titan Krios 300kV with a K3 Direct Electron Detector.

**Results:** The TSHR-ECD-M22^TM^ structure was solved to a global resolution of 3.7Å. A model was built using the previously solved TSHR-K1-70^TM^ cryo-EM structure and the M22^TM^-TSHR-LRD crystal structure.

M22^TM^ and TR1402, both potent agonists and K1-70^TM^ a potent antagonist all form strong interactions with TSHR-ECD residues.

Analysis of the interactions of the three ligands with the TSHR-ECD showed that M22^TM^ interacted strongly with TSHR R255 while neither K1-70^TM^ nor TR1402 had interacting residues. This is in good agreement with the observation that mutation of TSHR R255 prevents stimulation of the receptor by M22^TM^ and stimulating autoantibodies in patient sera while having no effect on K1-70^TM^ or TSH activity.

Furthermore, interactions were observed in the cryo-EM structure between TR1402 and TSHR residue Y385 while neither M22^TM^ nor K1-70^TM^ had interacting residues, in good agreement with experimental evidence that mutation of sulphated TSHR residue Y385 decreases receptor activation by TSH and TSH superagonists but not by M22^TM^ or thyroid stimulating autoantibodies in patient sera.

**Conclusions:** The different interactions seen in the structures of TSHR-ECD complexed with M22^TM^, K1-70^TM^ or TR1402 are in good agreement with the differences observed in the functional characteristics of the three ligands, supporting the accuracy of the cryo-EM structures.

## OR5 Systematic review of mortality and long-term major cardiovascular events (MACE) following different treatment approaches for hyperthyroidism

### Lauren Quinn^1^, Harita Yelamanchili^2^, Kristien Boelaert^2^, Barbara Torlinska^2^

#### ^1^Institute of Immunology and Immunotherapy, University of Birmingham, B15 2TT, UK; ^2^Institute of Applied Health Research, University of Birmingham, B15 2TT, UK

##### **Correspondence:** Lauren Quinn (l.quinn.1@bham.ac.uk)

*Thyroid Research 2024*, **17(S1):**OR5

**Objectives:** Hyperthyroidism affects up to 3% of the population and is associated with arrhythmias, which predispose to myocardial infarction, stroke and pulmonary embolism. Multiple studies indicate that all-cause and cardiovascular mortality are higher in patients with hyperthyroidism compared to the general population. However, associations between treatment modalities for hyperthyroidism and long-term health outcomes remain unclear. This study aims to analyse the literature and establish whether any of these treatments revert the long-term effects of hyperthyroidism.

**Methods:** Medline and Embase were searched for studies on the effects of different treatments for hyperthyroidism (antithyroid drugs (ATD), radioactive iodine (I-131) and thyroid surgery) on mortality and major adverse cardiovascular events (MACE) in adult patients. References and citations of selected full-text studies were screened. Two reviewers independently assessed eligibility and extracted the data. Bias was assessed with the Ottawa-Newcastle Scale. Outcome data were pooled to compare pairwise hazard rates (HR) using random effects (REML). The study forms part of the network meta-analysis registered in Prospero at the Centre of Reviews and Dissemination (CRD42024524000).

**Results:** The included studies consisted of large routinely collected cohorts at the national or regional level (Wales, Taiwan, Sweden, Hong Kong, Finland and England), together comprising data on 294,738 patients with average follow-up ranging from 1.5-10.5 years. There was only one study comparing the effects of treatment to the matched background population, showing an increased mortality risk after ATD and I-131 not resolving hyperthyroidism, but not when I-131 induced hypothyroidism within 1 year. When treatment approaches were compared pairwise (5 studies), surgery significantly improved survival when compared with ATD (HR=0.43 [95%CI: 0.30-0.62]). The survival after surgery (0.66 [0.41-1.06]) or ATD (0.85 [0.66-1.09]) compared with I-131 therapy was not statistically different. Risk of MACE (4 studies) was increased in all treatment groups when compared to matched controls without hyperthyroidism (ATD: 1.72 [1.23-2.41], I-131: 1.85 [1.17-2.98], surgery: 1.11 [1.03-1.19]). Pairwise comparisons of treatments (3 studies) indicated the highest reduction of MACE risk when surgery was compared to ATD (0.52 [0.25-1.07]), but the effect did not reach statistical significance (*P*=0.08). I-131 did not significantly affect MACE risk compared with ATD or surgery (ATD: 0.89 [0.61-1.29], surgery: 0.65 [0.25-1.68]).

**Conclusion:** Surgery was associated with improved outcomes in mortality and MACE when compared with medical treatment. Current data comparing long-term health consequences following different treatment approaches for hyperthyroidism are sparse. Further studies are needed to support informed decision-making when choosing the optimal therapeutic approach for hyperthyroidism.

## L2 BTF research award update: Longitudinal changes in thyroid function in survivors of the Whickham cohort – association with health

### Salman Razvi^1^, Marian Ludgate^2^

#### ^1^Newcastle University, Newcastle upon Tyne, UK; ^2^Cardiff University, Cardiff, UK

##### **Correspondence:** Salman Razvi (salman.razvi@newcastle.ac.uk)

*Thyroid Research 2024*, **17(S1):**L2

**Background:** Several cohort studies have assessed longitudinal changes in thyroid function but their results differ from one another. It is also unclear whether changes in thyroid function with age, if present, are associated with other markers of health and disease.

**Methods:** Survivors of the Whickham cohort had their thyroid function (TSH, FT4 and FT3) and TPOAb evaluated in 2008-2012 and reassessed again in 2016-2019 using the same assays (Roche e-cobas). Participants with known thyroid diseases or on medications affecting thyroid function were excluded. Detailed medical history was obtained at both study visits and markers of muscle strength (hand grip strength) and mobility (timed up-and-go test) were assessed at the second visit. Paired *t-tests* were utilised to compare thyroid function and linear regression analyses were used to assess predictors of changes in thyroid function.

**Results:** Over a mean interval of 7.6 years, 204 participants attended both study visits (mean age at baseline 77 years, % female, % smokers, and mean BMI 27.8 kg/m^2^). There was a small but significant increase in serum TSH level of 0.36 mU/L, FT4 of 0.23 pmol/L, and a reduction in FT3 by -0.1 pmol/L, over the study interval. Both upper and lower limits of the TSH reference interval (2.5^th^ and 97.5^th^ percentile) changed over the study period from 0.05 - 4.74 mU/L to 0.01 - 6.2 mU/L. The number of comorbidities significantly predicted a reduction in TSH; otherwise, no other factors were significantly associated with changes in thyroid function including age, sex, smoking status, BMI, or TPOAb status. There was no significant change in TPOAb levels. Hand grip strength was not associated with changes in thyroid function. The timed up-and-go test was associated with changes in FT4 levels: for each 1 pmol/L rise, there was a 0.04 (0.01 – 0.07) seconds multivariable-adjusted increase.

**Conclusions:** This study has confirmed that thyroid function parameters demonstrate minor changes with age. These changes do not appear to be associated with markers of muscle strength. The observed negative relationship between FT4 and mobility deserves further assessment. Overall, thyroid function changes with time and age-appropriate reference ranges should be considered in routine clinical practice to reduce the risk of unnecessary treatment.

## PO1 Isolating human C cells by FACS for downstream -omics analysis to better understand the development of MTC in MEN2 patients

### Jessica Fear^1^, Guillaume E. Desanti^2^, Katie Brookes^1^, Naomi Richardson^3^, Caitlin E.M. Thornton^1^, Ling Zha^1^, Vicki E. Smith^1^, Martin L. Read^1^, Renuka P. Dias^1,4^, Christopher J. McCabe^1^

#### ^1^Institute of Metabolism and Systems Research (IMSR), and Centre of Endocrinology, Diabetes and Metabolism (CEDAM), University of Birmingham, Birmingham, UK; ^2^Institute of Immunology and Immunotherapy, University of Birmingham, Birmingham, UK; ^3^Centre for Liver and Gastrointestinal Research & NIHR Birmingham Liver Biomedical Research Unit, Institute of Biomedical Research, Institute of Immunology and Immunotherapy, University of Birmingham, Birmingham, UK; ^4^Department of Endocrinology and Diabetes, Birmingham Women and Children’s Hospital, Birmingham, UK

##### **Correspondence:** Jessica Fear (j.fear.1@bham.ac.uk)

*Thyroid Research 2024*, **17(S1):**PO1

**Background:** Multiple endocrine neoplasia type 2 (MEN2) is an inherited condition caused by RET proto-oncogene mutations and is responsible for approximately 20% of medullary thyroid cancer (MTC) cases. Predicting the onset of MTC in MEN2 patients is difficult and can vary between individuals in the same family with the same RET mutation. Enhanced molecular understanding of C cells via multi-omics approaches will be key to addressing the heterogeneity of RET mutational impacts. Previously, rat C cells have been isolated using Fluorescence-activated cell sorting (FACS), but this has not been reported for human C cells.

**Aims:** To establish a technique for isolating C-cells from fresh human thyroid tissues for -omics analysis to determine the individual risk factors influencing MTC development in MEN2 patients.

**Methods:** FACS was performed on fresh human primary thyroid tissues and mixed thyroid cell line populations to sort C cells, based on staining for the intracellular markers calcitonin and chromogranin A. TaqMan RT-PCR was used to measure the expression of calcitonin in FACS sorted cells to appraise their identity.

**Results:** FACS validations were initially performed on (i) two adult human primary thyroid samples, and (ii) mixed thyroid cell line populations containing 5% MTC-derived TT cells and 95% 8505C and TPC1 cells. Calcitonin, chromogranin A and double positive populations were isolated using FACS. Having established appropriate experimental conditions, FACS was subsequently performed on a primary human paediatric thyroid tissue sample which was successfully sorted into calcitonin-positive (24,214 cells), chromogranin A-positive (2,796 cells), double-positive (1,208 cells) and autofluorescent (21,860 cells) populations. TaqMan RT-PCR confirmed the expression of calcitonin in the sorted cell subgroups.

**Conclusions:** We designed a FACS protocol for isolating C cells for -omics analysis with future potential applications in understanding and improving personalised risk stratification in MEN2.

## PO2 Thyroid hormone profiles in individuals on non-standard thyroid hormone replacement

### Peter Taylor^1^, Aneela Arooj^1^, Stephanie Hanna^1^, Suhani Bahl^1^, Vinay Eligar^2^, Zubair Muhammad^3^, Mike Stedman^4^, Lakdasa Premawardhana^1^, Onyebuchi Okosieme^1^, Adrian H Heald^5^, Colin Dayan^1^

#### ^1^Thyroid Research Group, Systems Immunity Research Institute, Cardiff University School of Medicine, Cardiff, UK; ^2^Imperial College London Diabetes Centre, Abu Dhabi, UAE; ^3^Res Consortium; ^4^University of Manchester, Manchester, UK

##### **Correspondence:** Peter Taylor (taylorpn@cardiff.ac.uk)

*Thyroid Research 2024*, **17(S1):**PO2

**Background and aims:** Levothyroxine(T4) monotherapy is the mainstay of thyroid hormone replacement. However, a growing number of patients utilise alternatives such as combination thyroid hormone replacement, liothyronine (T3) monotherapy and desiccated thyrfThe proto-oncogene pituitaryoid extract (DTE). Biochemical monitoring is more complex in this therapeutic situation, so we explored thyroid hormone profiles in these patients over 8 hours.

**Methods:** We performed hourly blood tests (08.30-16.30pm) to assess thyroid stimulating hormone (TSH), freeT3 (FT3) and Free T4(FT4) levels in 44 individuals (*N*=17 combination thyroid hormone replacement (T4+T3), *N*=12 on T3 monotherapy and *N*=15 on DTE). Area under the curve (AUC) analysis was performed and odds of having a very low TSH) (<0.05mU/l) and completely suppressed TSH (<0.02mU/l) at 08:30 were analysed with adjustment for age.

**Results:** T3 monotherapy and DTE had higher AUC FT3 levels and lower AUC FT4 levels than combination thyroid hormone replacement. Highest FT3 levels were seen with T3 monotherapy. Combined T3 and T4 dose, T3 dose, and T4 dose were not associated with increased odds of a very low or a completely suppressed TSH. AUC FT3 was associated with increased odds of very low TSH OR=2.95 (95%CI 1.45-6.03) *p*=0.003 and a completely suppressed TSH OR=2.21 (95%CI 1.12-4.38) *p*=0.02. Peak FT3 was associated with increased odds of very low TSH OR=2.51 (95%CI 1.24-5.05) *p*=0.01 and completely suppressed TSH OR=2.31 (95%CI 1.13-4.70) *p*=0.02. No association was seen with AUC FT4 or peakFT4 level vs TSH. Any FT3 level above 7.0pmol/l (as seen particularly with LT3) was associated with increased odds of a very low TSH OR=11.7 (95%CI 1.23-111) *p*=0.03.

**Discussion:** FT3 levels have a greater negative impact on TSH levels than FT4 levels. This impedes maintenance of normal TSH levels in individuals on non-standard thyroid hormone replacement. Notably peak FT3 levels appear to be more important in influencing TSH levels than total T3 dose. Taken together this suggests that strategies to reduce peak T3, such as slow release T3, should be investigated as a path to enabling moderate T3 doses without substantially suppressing TSH.

## PO3 Proto-oncogene PBF regulation of cell adhesion and motility

### Selvambigai Manivannan^1^, Merve Kocbiyik^1^, Davina Banga^1^, Afshan Afzal^1^, Ling Zha^1^, Katie Brookes^1^, Hannah R Nieto^1^, Steve Thomas^2^, Martin L Read^1^, Chris J McCabe^1^, Vicki E Smith^1^

#### ^1^Institute of Metabolism and Systems Research (IMSR), University of Birmingham, UK; ^2^Institute of Cardiovascular Sciences (ICVS), University of Birmingham, UK

##### **Correspondence:** Selvambigai Manivannan (s.manivannan@bham.ac.uk)

*Thyroid Research 2024*, **17(S1):**PO3

**Background and Aims:** The proto-oncogene pituitary tumor-transforming gene (PTTG)-binding factor (PBF/PTTG1IP) is upregulated in thyroid cancer and associated with tumour progression. PBF potently induces thyroid cancer cell motility via Src kinase phosphorylation. We have recently shown that PBF is also required for physiological mouse embryonic fibroblast (MEF) motility. Pbf-knockout (KO) MEFs have significantly reduced migration and invasion compared with wild-type (WT) MEFs. Phosphoproteomic and RNA-Seq analyses revealed that PBF upregulation in Nthy-ori 3-1 thyroid cells altered expression and phosphorylation of key adhesion proteins. We hypothesised that PBF physiologically regulates cell adhesion, and its oncogenic expression promotes thyroid cancer cell motility via altered adhesion. This study aimed to further elucidate the regulation of cell adhesion by PBF.

**Methods:** We utilised Pbf-KO MEFs and CRISPR/Cas9-mediated PBF-KO TPC-1 human papillary thyroid carcinoma cells in the analysis of cell adhesion and spreading on fibronectin-coated plates.

**Results:** Cell adhesion assays demonstrated that Pbf-KO MEFs exhibited markedly decreased cell-substrate adhesion compared with Pbf-WT MEFs. We then assessed focal adhesions (FAs), the large protein complexes that link the cell cytoskeleton to the extracellular matrix. Immunofluorescence staining of focal adhesion kinase (FAK), vinculin and paxillin revealed fewer and shorter FAs located predominantly around the periphery of Pbf-KO MEFs, in comparison with Pbf-WT MEFs, which displayed numerous, elongated FAs along actin fibres throughout the cells. TPC-1 PBF-KO cells also demonstrated decreased cell-substrate adhesion, as well as reduced cell spreading. In support of this, LifeAct-GFP live cell imaging suggested that both PBF-KO MEFs and TPC-1 cells had impaired cell spreading and loss of orientation.

**Conclusions:** Taken together, these findings provide new mechanistic insights into the regulatory role of PBF in thyroid cancer cell motility through cell adhesion dynamics. These findings also highlight potential avenues to therapeutically target PBF regulated pathways in tumorigenesis.

## PO4 A new TSH receptor preparation for the study of TSH receptor autoimmunity

### L. Allen, S. Reddington, B. Harteveld, W. Dowler, P. Sanders, A. Sullivan, S. Young, M. Holly, J. Sanders, B. Rees Smith

#### FIRS Laboratories, RSR Ltd, Parc Ty Glas, Llanishen, Cardiff, UK

##### **Correspondence:** L. Allen (firs@rsrltd.eclipse.co.uk)

*Thyroid Research 2024*, **17(S1):**PO4

**Background and Aims:** Preparations of pure, stable TSH receptor (TSHR) are important tools in studies of TSHR autoimmunity and we now describe such a preparation consisting of TSHR aa 22-260 with stabilising mutations (TSHR260-STABL^TM^).

**Methods:** TSHR260-STABL^TM^ was expressed in insect cells and purified by ion exchange and affinity chromatography. Purity was confirmed using analytical size exclusion chromatography, SDS-PAGE and Western blotting. Binding of TSHR stimulating monoclonal autoantibodies M22^TM^ and K1-18^TM^ and blocking autoantibody K1-70^TM^ to TSHR260-STABL^TM^ was assessed following biotin labelling, radiolabelling, and coating onto ELISA wells. The ability of plastic tubes coated with TSHR260-STABL^TM^ to absorb patient sera TSHR autoantibodies (TRAb) was investigated.

**Results:** TSHR260-STABL^TM^ interacted well with TSHR monoclonal autoantibodies M22^TM^, K1-18^TM^ and K1-70^TM^ in a bridge ELISA based on TSHR260-STABL^TM^-biotin and plate wells coated with full length TSHR.

In the assay as little as 0.3 U/L of NIBSC 08/204 (2.5 ng/mL of M22^TM^ IgG) was readily detectable. Also, 101/103 (98 %) of untreated Graves’ disease patients gave positive results in the assay.

TSHR260-STABL^TM^ coated tubes were able to absorb TSHR autoantibodies in 54 of 55 sera, providing a convenient specificity test for various TSHR autoantibody assays and proof of principle for therapeutic immunoabsorption applications.

Pure TSHR260-STABL^TM^ activity was tested following incubation at 37 °C for 14 days and demonstrated no loss of activity. Similarly, TSHR260-STABL^TM^ – biotin stored at 37 °C for up to 8 weeks demonstrated excellent stability with no loss of activity in detecting M22^TM^.

**Conclusions:** TSHR260-STABL^TM^ and TSHR260-STABL^TM^-biotin are TSH receptor preparations which react well with TSHR autoantibodies. These stable materials should be valuable tools in studies of humoral and cellular autoimmunity to the TSHR. Also, they should be useful in therapeutic interventions such as specific immunoabsorption of TSHR autoantibodies.

## PO5 Micro RNAs in the core arsenal of molecular paradigms in papillary thyroid cancer modalities

### Rashida Khan^1^, Ruqia Mehmood Baig^2^

#### ^1^Institute of Biomedical & Genetic Engineering, (IBGE) Islamabad, Pakistan; ^2^PMAS-Arid Agriculture University Rawalpindi, Pakistan

##### Correspondence: Rashida Khan (dr.rashidakhan85@gmail.com)

*Thyroid Research 2024*, **17(S1):**PO5

**Background:** Thyroid cancer is considered to exist among most common cancers and at the same time it is the most recurrent malignancy of the endocrine system. The most frequent type of thyroid cancer is papillary thyroid cancer (PTC), which contributes more than 80% globally & prevalent in females of Eastern and Western Asia, America and Iceland. The genetic elements known as MicroRNAs (miRNAs), endogenous non-coding RNAs operating as post-transcriptional regulators involved in development, proliferation and differentiation. miRNAs are gaining fame as druggable biomarkers and clinical management of neoplasm. The momentous stackeholding of miRNAs by virtue of gene expression variations in the cancer microenvironment has been witnessed in the PTC onset, amplification and apoptosis. The growing body of knowledge highlights the modifiable play at the miRNA level harbors potential in lessening the perpetuation of the disease with safe handlers. The genetic information leads to a big highway which can replace the unified yardstick to tailor PTC with the more targeted personalized disease treatment by monitoring the disease risk and aggression modalities.

**Objectives:** The study aims to speculate the characteristic involvement of expression level changes in the miRNA genes miRNA-146b and miRNA-181b as tangible biomarkers for papillary thyroid cancer.

**Methodology:** The present study was conducted on the PTC in Pakistan, a genetically less explored South Asian country. Specimen of cancer tissue, normal samples and multi nodular goiter (MNG) samples from patients were collected. The anthropometric and clinical parameters of patients were recorded after informed consent. Total RNA was isolated and cDNA was synthesized. Gene expression profile for miRNA-146b and miRNA-181b was done by quantitative Real-Time PCR. Relative gene expression was identified as fold change and mutational deregulations were checked through DNA Sanger sequencing showing the involvement of these miRNAs in PTC.

**Results:** The statistically significant relative expression of genes miRNA-146b; 5 to 20 folds and miRNA-181b; 4-60 folds were observed in PTC in comparison to MNG and healthy tissue specimens. Similarly, rare genotypes and alleles were observed significantly in PTC tissue samples compared to controls and MNGs.

**Conclusion:** The boosted gene expression of the miRNA genes miRNA-146b and miRNA-181b manifests the plausible misregulations in deployment of these molecular musketeers as foes in PTC. This forged maladaptation of the miRNA-146b and miRNA-181b in the cancer microenvironment may warrant analytically, therapeutically and genetically surmountable miRNA targets for PTC clinical management prevention.

## PO6 Utilisation of near infrared autofluorescence in parathyroid identification during thyroidectomy: a meta-analysis of randomised controlled trials

### Azfar Javed, Abdullah Alburaiki, Neil Sharma, Mriganka De, George Garas, Ijaz Ahmad, Paul Nankivell, Anita Sonsale, Jonathan Fussey, Keshav Kumar Gupta

#### Queen Elizabeth Hospital, University Hospital Birmingham NHS Foundation Trust, Birmingham, UK

##### **Correspondence:** Azfar Javed (azfar.javed1@nhs.net)

*Thyroid Research 2024*, **17(S1):**PO6

**Objective:** Unintentional parathyroid gland resection during total thyroidectomy can result in permanent hypoparathyroidism and the need for lifelong replacement therapy. Near infrared autofluorescence (NIRAF) imaging aids in the intraoperative identification and preservation of the parathyroid glands. Over the last five years, several randomised controlled trials (RCTs) have investigated NIRAF use in thyroid surgery for the prevention of hypoparathyroidism. This systematic review and meta-analysis aims to review NIRAF’s effectiveness in intraoperative parathyroid identification and prevention of post-operative hypoparathyroidism.

**Methods**: This meta-analysis was undertaken aligning with PRISMA guidelines following a protocol pre-registered on PROSPERO. Online searches of Medline and Embase databases, as well as grey literature, were performed up to January 2024. Included articles were RCTs that studied the use of NIRAF versus dissection with no intraoperative aids in total thyroidectomy and included intra-operative findings and post-operative outcomes. Meta-analysis was performed using a random-effects model. Quality assessment was performed using the Cochrane Risk of Bias and GRADE assessment tools. Primary outcomes were parathyroid gland identification, post-operative hypocalcaemia, and permanent hypoparathyroidism.

**Results:** Seven trials were included in the final meta-analysis, comprising 1,384 patients. Patients undergoing thyroidectomy using NIRAF had a reduced risk of post-operative hypocalcaemia (OR 0.56, 95% CI: 0.36-0.89, *p*=0.01). The pooled meta-analysis showed a non-significantly reduced rate of persistent hypoparathyroidism in the NIRAF group (OR 0.50, 95% CI: 0.24-1.07, *p*=0.07).

**Conclusion:** NIRAF use in thyroidectomy surgery reduces the risk of post-operative hypocalcaemia. However, no significant reduction in permanent hypoparathyroidism was identified.

## PO7 Delayed onset of extrathyroidal manifestations of Graves’ disease following radioiodine treatment

### Nwe Aung, Amina Al-Qaysi, Chitrabhanu Ballav

#### Buckinghamshire NHS Trust, UK

##### **Correspondence:** Nwe Aung (nwe.aung@nhs.net)

*Thyroid Research 2024*, **17(S1):**PO7

**Background:** Extrathyroidal manifestations of Graves’ disease (GD) include ophthalmopathy, dermopathy and acropachy. Thyroid acropachy presentation occurs in less than 1% of individuals with GD. It typically coexists with ophthalmopathy and thyroid dermopathy, serving as a severity indicator of these conditions. Autoantibody levels in autoimmune thyroid disease like GD may transiently increase following Radioiodine (RI) treatment but decline in the long term. We report a patient who presented with thyroid acropachy, dermopathy, and orbitopathy manifesting multiple years following RI treatment for GD.

**Case-report:** A 48-year-old gentleman presented twenty-seven years after receiving RI for recurrent GD followed by Levothyroxine supplement for post-RI hypothyroidism starting months after treatment. He had painless clubbing of his hands and toes. He had a non-tender lump affecting his right big toe. He had developed symmetrical bilateral proptosis consistent with Grave’s orbitopathy and preserved vision seven years after RI.

MRI orbits confirmed bilateral proptosis with normal signal over the extraocular muscles. X-ray of the right big toe revealed soft tissue swelling overlying the proximal phalanx of the great toe with normal underlying bone. He was euthyroid (TSH 0.7 mIU/L, range 0.35 - 4.94, Free T4 17.2 pmol/L, range 9.01 - 19.05, and Free T3 3.8 pmol/L, range 2.63 - 5.7) on 150 mcg per day of Levothyroxine. His TRAB was > 30 IU/mL, range 0-0.5. He was referred to the dermatology department for cosmetic concerns over dermopathy.

**Conclusion:** TRAB may rise after radioiodine therapy but declines long-term. The reported case study demonstrates that levels may be significantly high and present with extrathyroidal features several years after RI treatment.

The patient has given consent for publication of this abstract.

## PO8 Do we meet the proposed quality performance indicators from the scottish thyroid cancer network? – Experience of a Scottish Tertiary Centre

### Shao Hao Alan Yap, Alex J Graveling, Prakash Abraham

#### JJR Macleod Centre for Diabetes & Endocrinology, Aberdeen Royal Infirmary, Aberdeen, UK

##### **Correspondence:** Shao Hao Alan Yap (alan.yap@nhs.scot)

*Thyroid Research 2024*, **17(S1):**PO8

**Background:** Differentiated thyroid cancer (DTC) is the most common endocrine malignancy. The Scottish Thyroid Cancer Network endeavours to standardize the investigation and treatment of thyroid cancer patients across Scotland. Quality Performance Indicators (QPIs) are being developed to assess performance in treating thyroid cancer patients.

**Aims:** This study evaluates whether a Thyroid Cancer treatment centre meets specific QPIs (see table below for details).

**Methods:** Retrospective study conducted in a tertiary endocrine service, focusing on patients who attended between May 2015 and November 2022. The study included patients diagnosed with DTC and examined data on their surgery dates, Radioiodine remnant Ablation (RRA) treatment doses and dates, and follow-up appointments involving Thyroglobulin/ Thyroglobulin antibody levels and neck ultrasound assessments.

**Results:** Out of 116 patients identified, 24 received 1.1 GBq RRA, 85 received 3.7 GBq RRA, 6 received 5.5 GBq RRA, and 1 received 7.4 GBq RRA. The outcomes of each QPI are shown in the table.

**Conclusion:** We are not currently meeting these QPIs, with delays partly due to the COVID-19 pandemic (treatment & radiology delays, patients choosing low dose to reduce delay). This data allows examination of the rate limiting steps that would allow us to make provisions to achieve these QPIs. These findings offer valuable insights into adherence to treatment guidelines and the efficacy of current management approaches for DTC patients.

**Table:** 

QPI	Description	Target	Result
4	The proportion of DTC patients having completion thyroidectomy within 3 months of initial lobectomy.	70%	38(67.9%)
5	The proportion of patients with T4 and/or N1b or metastatic DTC who have undergone thyroidectomy and receive 3.7GBq RRA	90%	43(89.5%)
6	The proportion of DTC patients receiving 3.7GBq RRA within 3 months post-thyroidectomy.	70%	21(24.7%)
7	The proportion of DTC patients receiving RRA with Dynamic Risk Stratification (DRS) within 12 months post-treatment	90%	58(50.4%)

## PO9 Safety of outpatient low dose 1.1 GBq Radioiodine Remnant Ablation (RRA) treatment for differentiated thyroid cancer (DTC)

### Shao Hao Alan Yap^1^, Fergus McKiddie^2^, Alex J Graveling^1^, Prakash Abraham^1^

#### ^1^JJR Macleod Centre for Diabetes & Endocrinology, Aberdeen Royal Infirmary, Aberdeen, UK; ^2^Nuclear Medicine Department, Aberdeen Royal Infirmary, Aberdeen, UK

##### **Correspondence:** Shao Hao Alan Yap (alan.yap@nhs.scot)

*Thyroid Research 2024*, **17(S1):**PO9

**Background:** Long-term follow-up of lower-risk differentiated thyroid cancer (DTC) patients (T1-T3 N0/1) confirmed similar recurrence rates following 1.1GBq or 3.7GBq radioiodine remnant ablation (RRA). While recipients of 3.7GBq RRA require inpatient treatment, outpatient administration of 1.1GBq is possible. This promises improved patient experience and cost reduction. Dose-rate measurements post RRA and extrapolation of decline in residual activity allow personalised tailoring of radiation protection precautions, often enabling patients to resume normal activities sooner.

**Aims:** Outpatient administration of 1.1GBq RRA was compared with inpatient administration of both 1.1GB and 3.7GBq RRA. Time for the dosimeter to fall below 1.5 microSieverts (mSv) was calculated. Below this level, all radiation protection precautions are lifted.

**Methods:** Retrospective study from a Scottish Tertiary centre, focusing on DTC patients attending between 2018-2023 who underwent RRA after complete thyroidectomy. RRA 1.1GBq was administered as inpatient or outpatient, 3.7GBq was administered as inpatient. Dose rates from thermoluminescent dosimeters were extrapolated to calculate time to fall below 1.5mSv.

**Results:** A total of 33 patients, aged 20-86 years, received 1.1GBq RRA (11 outpatients and 22 inpatients) while 35 patients, aged 25-87 years, received 3.7GBq RRA as inpatients. The mean time for the dosimeter to reach <1.5mSv was 55.9 hours for inpatients and 64.1 hours for outpatients following 1.1GBq RRA, and 97.4 hours following 3.7GBq RRA. 85%(28) of 1.1GBq RRA patients achieved this in 72 hours, while 97%(32) did so within 96 hours. For 3.7GBq RRA patients, 11.4%(4) achieved this in 72 hours, while 60%(21) did so within 96 hours.

**Conclusion:** Outpatient administration of low-dose 1.1 GBq RRA is safe and comparable with inpatient treatment for DTC patients. There is limited literature about dosimetry reading post RRA, particularly following the introduction of Thyrogen. The rapid clearance is reassuring and merits further study to see if radiation protection restrictions can be eased earlier.

## PO10 Evaluating pregnancy outcomes, such as stillbirth and premature birth, along with infant health factors -low birth weight and the necessity for intensive care in women diagnosed with thyroid cancer

### Salomea Guchmazashvili^1^, Maia Kereselidze^2^, Tinatin Manjavidze^2^

#### ^1^University of Georgia School of Health Sciences, Tbilisi, Georgia; ^2^Department of Medical Statistics, National Center for Disease Control and Public Health, University of Georgia School of Health Sciences, Tbilisi, Georgia

##### **Correspondence:** Salomea Guchmazashvili (saloguchmazashvili@gmail.com)

*Thyroid Research 2024*, **17(S1):**PO10

***Background and Objectives:*** The prevalence of thyroid cancer is rapidly increasing globally, expected to rank as the fourth most common cancer by 2030. This surge significantly impacts women affecting their reproductive health due to anticancer therapies. The study aims to evaluate pregnancy outcomes and infant health in women diagnosed with thyroid cancer. Study objectives include determining the association between thyroid cancer and pregnancy outcomes such as premature birth, stillbirth, alongside examining newborn health.

***Methods:*** Retrospective cohort study was conducted which identified women diagnosed with thyroid cancer between 2015 and 2023 from a population-based cancer registry, in total 6,500. Data were merged with the Georgian Birth Registry (2016-2023), identifying a study group of 1,200 women with thyroid cancer who had either delivery or abortion during the study period. A control group comprised non-cancer women without any history of thyroid disease or other chronic conditions. Statistical analysis – chi square test was conducted using SPSS.

***Results:*** An analysis of data from the cancer population register and the Georgian Birth Register, covering 238,000 women both with and without thyroid cancer, revealed following results: out of 293 women with thyroid cancer, less than 0.7% gave birth at 36 weeks of gestation or earlier. Among 581 women with thyroid cancer, only 0.9% experienced stillbirths. Meanwhile, 10.4% of 587 women with thyroid cancer delivered newborns weighing less than 2500 grams (as per WHO classification), indicating low birth weight. Additionally, 11.3% of newborns from 372 women with thyroid cancer required intensive care unit (ICU) admission.

***Conclusions:*** The study highlights the critical association between thyroid cancer in women and adverse neonatal outcomes, particularly low birth weight and the necessity for intensive care post-delivery. These findings underscore the importance of specialized care for pregnant women with thyroid cancer to mitigate risks to newborn health, although no significant links to premature birth or stillbirth were observed.

## PO11 Tuberculosis thyroiditis: a case report

### I. Boughazi^1^, I. Benaceur^2^

#### ^1^Department of Endocrinology and diabetes, Chelsea and Westminster Hospital, London, UK; ^2^Douera cytology Laboratory, Algiers, Algeria

##### **Correspondence:** I. Boughazi (imaneboughazi2020@gmail.com)

*Thyroid Research 2024*, **17(S1):**PO11

**Introduction:** Despite the rise in extrapulmonary tuberculosis in Algeria, the occurrence of tuberculosis in the thyroid remains a rarity. Which can be attributed to the thyroid's resistance to Mycobacterium Tuberculosis. About 200 cases have been reported globally, the clinical manifestations are nonspecific, with caseating necrosis found in cytological studies being the key factor for confirming diagnosis. And the Mycobacterium Tuberculosis is rarely detected in cultures.

**Case-report:** A 50-year-old patient with well-managed type 2 diabetes was referred to the endocrine clinic in Algiers, for management of thyroid nodules. Clinically, the patient exhibited asthenia, but did not show signs of weight loss, fever or night sweats. Both clinical and biological assessments indicated euthyroidism.

A cervical ultrasound revealed thyroid nodules in the right lobe, within a eutrophic thyroid gland. The largest nodule was inhomogeneous hypoechoic, with a long axis measuring 18mm, and was classified as EUTIRADS4A. Cytology results suggested subacute granulomatous thyroiditis with caseating necrosis, indicative of tuberculous thyroiditis.

The patient was then referred to the tuberculosis control department for further investigations. The diagnosis of extrapulmonary thyroid tuberculosis without any other location was confirmed, and a 6-month course of anti-tuberculosis treatment was initiated. The course of treatment was well-tolerated by the patient, with clinical and biological euthyroidism.

A post-therapeutic ultrasound showed an increase in nodular volume to 21mm, which was reclassified as TIRADS3 due to cystic degeneration and haemorrhagic changes observed in the cytology. These findings were consistent with the healing process of tuberculous lesions.

**Conclusion:** The presentation of thyroid tuberculosis is often nonspecific, and the diagnosis is usually incidental, as was the case with our patient. However, most reported cases have been diagnosed through a histological study of surgical specimens. Recovery is typically achieved through anti-tuberculosis treatment.

The patient has given consent for publication of this abstract.

## PO12 Pregnancy Reference Range Study (PREGRRS): trimester-specific reference ranges using Abbott Alinity immunoassays for thyroid status in pregnancy

### M. Abdel-Malek^2,3^, R.V. Scott^1,2^, J. Zhu^3^, J. Tan^3^, D. Thayabaran^3^, R. Valaiyapathi^3^, P. Padam^3^, E. Jackson^3^, S.C. Barnes^3^, H. Fourie^4^, M. Al-Memar^4^, C. Kyriacou^4^, M. Nimura^4^, T. Bourne^4,5,6^, N.G. Martin^3^, R. Agha-Jaffar^7^, B. Jones^8^, B. Khoo^9^, T.M-M. Tan^2,3^

#### ^1^Department of Endocrinology, Chelsea & Westminster Hospital, London, UK; ^2^Department of Metabolism, Digestion & Reproduction, Imperial College London, London, UK; ^3^Blood Sciences, North West London Pathology, Charing Cross Hospital, London, UK; ^4^Tommy’s National Centre for Miscarriage Research, Queen Charlotte’s and Chelsea Hospital, Imperial College London, London, UK; ^5^Department of Development and Regeneration, KU Leuven, Leuven, Belgium; ^6^Department of Gynaecology, University Hospital Leuven, Leuven, Belgium; ^7^Department of Endocrinology, Imperial College Healthcare NHS Trust, London, UK; ^8^Department of Obstetrics & Gynaecology, Imperial College Healthcare NHS Trust, London, UK; ^9^Department of Endocrinology, Division of Medicine, University College London, Royal Free Campus, London, UK

##### **Correspondence:** Mariana Abdel-Malek (m.abdel-malek@nhs.net)

*Thyroid Research 2024*, **17(S1):**PO12

**Introduction:** The physiological changes in maternal thyroid hormone levels are well-established. In early gestation, human chorionic gonadotrophin elevation exhibits thyrotropic activity which can lead to higher circulating free thyroxine (fT4) and thyroid stimulating hormone (TSH) suppression. Oestrogen increases thyroxine binding globulin (TBG) production, and subsequently total thyroxine (tT4), causing fT4 to vary throughout pregnancy. This is the first study to produce trimester-specific reference ranges for thyroid function in a diverse multi-ethnic pregnant population using the Abbott Alinity analyser in line with American Thyroid Association guidelines.

**Methods:** Blood samples were collected from 725 healthy pregnant people receiving antenatal care at Queen Charlotte’s hospital. Participants without history of thyroid dysfunction or positive thyroid peroxidase antibodies were included. TSH, fT4 and fT3 measurement was performed by a two-step immunoassay using chemiluminescent microparticle immunoassay (CMIA) technology on the Abbott Alinity platform. Urinary iodine was measured by inductively coupled plasma mass spectrometry (ICP-MS) on Agilent 7900 ICP-MS.

**Results:** Reference ranges were defined as the 2.5^th^-97.5^th^ centile (*n*=663). The mean age was 34 years (range 18-49) and 51% were white. Reference ranges for TSH were 0.06-2.73 mIU/l, 0.02-2.47 mIU/l and 0.41-2.80 mIU/l in the 1^st^, 2^nd^ and 3^rd^ trimesters. For fT4, the ranges were 9.9-15.3 pmol/l, 8.5-14.4 pmol/l and 7.6-12.3 pmol/l respectively. There were no significant differences in TSH, fT4 or fT3 ranges between white and non-white participants at any trimester. Participants were confirmed iodine replete: median urinary iodine concentration was 3.2 μmol/L, 4.6 μmol/L and 10.0 μmol/L in the 1^st^, 2^nd^ and 3^rd^ trimesters.

**Conclusion:** The gestation-specific thyroid reference ranges ascertained by our study are notably different from the manufacturer’s non-pregnancy ranges, and also vary across the trimesters. These ranges are important in more accurately diagnosing thyroid dysfunction in pregnancy as well as optimising the antenatal monitoring and management of those with pre-existing disease.

## PO13 Graves’ disease in pregnancy: a case series from preconception to postpartum

### Mariana Abdel-Malek^1,2^, Caroline Dawson^1^, Dri Choa^1^, Maud Hardy^1^, Lynee Sykes^1^, Jayne Terry^1^, Christina Yu^1^, Edward Mullens^1^, Jeremy Cox^1^, Stephen Robinson^1^, Rochan Agha-Jaffar^1^

#### ^1^Imperial College Healthcare NHS Trust, London, UK; ^2^Department of Metabolism, Digestion & Reproduction, Imperial College London, UK

##### **Correspondence:** Mariana Abdel-Malek (m.abdel-malek@nhs.net)

*Thyroid Research 2024*, **17(S1):**PO13

**Background:** The complications of uncontrolled thyrotoxicosis to pregnancy are well-known. Thionamide therapy carries its own risk for congenital malformations.

**Methods:** In this case series, we report 49 pregnancies in 29 women with thyrotoxicosis under joint antenatal care at St Mary’s Hospital: 26 women had underlying Graves’ disease and four were diagnosed antepartum.

**Results:** The median age at pregnancy was 32 years (range 19 to 42). There was no anti-thyroid drug (ATD) use for over a third of pregnancies in the preconception period (34.6%; *n=*17), 34.6% were on Carbimazole (*n=*17) and 22.4% on Propylthiouracil (*n=*11). The remaining women were on Levothyroxine after definitive treatment (thyroidectomy *n=*1; radioiodine *n*=2).

35.3% of those on no ATD were later commenced on thionamide therapy during pregnancy (*n*=2 Propylthiouracil; *n=*4 Carbimazole). Antenatal treatment remained the same in 69.4% of pregnancies. A switch between Carbimazole or Propylthiouracil occurred in five pregnancies and four thionamide-treated patients discontinued medication, of whom one had conceived on block and replace. Of the women who had a TSH receptor antibody checked (69.4%; *n*=34), 47.1% (*n*=16) were negative (<1.0unit/mL).

Miscarriage occurred in ten pregnancies (first-trimester *n=*9*;* second-trimester *n*=1). The mean gestational age was 37+4 (*n=*37) and 37.8% (*n=*14) of neonates were born preterm (≤37 weeks). Additional complications included one intrauterine death at 36 weeks, one neonatal death at 10 days and hypertension (including pre-eclampsia) in nine pregnancies. Of 23 women followed-up for at least one year postnatally, whom had not undergone definitive treatment, fourteen suffered a post-partum recrudescence of thyrotoxicosis (60.1%).

**Conclusions:** Thyrotoxicosis poses risks to a pregnancy even if an individual has controlled thyroid disease. This is potentially mitigated by definitive therapy in preconception which additionally avoids the risks of thionamide-associated congenital anomaly and relapse of thyrotoxicosis post-partum. Clinicians should address pregnancy plans in females of child-bearing status with Graves’ disease to optimise management.

## PO14 The relation between circulating free thyroxine (FT4) and thyroid stimulating hormone (TSH) levels is very different in people taking levothyroxine vs those who are on no thyroid hormone replacement

### A.H. Heald^1,2^, L.D. Premawardhana^3^, P.N. Taylor^3^, N. Chaudhury^4^, O.E. Okosieme^3^, M. Stedman^5^, C.M. Dayan^3^

#### ^1^The School of Medicine and Manchester Academic Health Sciences Centre; University of Manchester, Manchester, UK; ^2^Department of Endocrinology and Diabetes, Salford Royal Hospital, Salford, UK; ^3^Thyroid Research Group, Systems Immunity Research Institute, Cardiff University School of Medicine, Cardiff, UK; ^4^Department of Endocrinology and Diabetes, University Hospitals Coventry and Warwickshire, UK; ^5^Res Consortium, Andover, UK

##### **Correspondence:** A.H. Heald (adrian.heald@nca.nhs.uk)

*Thyroid Research 2024*, **17(S1):**PO14

**Introduction:** There continues to be much discussion around optimization of thyroid hormone status in hypothyroid individuals. The ideal therapeutic goal in hypothyroidism would be to restore clinical and biochemical euthyroidism via physiologic thyroid hormone replacement. This concept may seem straightforward, but there are subtleties that have only recently been recognized.

We here looked the way that FT4 and TSH related to each other in a large laboratory sample of people who underwent a check of thyroid function (TFT) split between those on levothyroxine replacement (monitoring test) and those who underwent a TFT check as a screening test for thyroid hormone imbalance (not on levothyroxine).

**Methods:** TFT test (FT4 and TSH) results were taken from the Salford Hospital (UK) laboratory system for 2009-2012. The request includes a tick box for ‘on levothyroxine’ (yes or no). Age and sex of patients was also available. To minimise comorbidity effects only samples taken in GP Practices were used and for untreated patients only those who had single tests results were used. For treated patients, the median value across all their results were used.

Cluster analysis compared the log(TSH) and FT4 values between treated and untreated population to highlight the % of treated patients achieving levels similar to the untreated population.

**Results:** Total data included 290,000 tests for 130,000 patients. However, the FT4/TSH results were used from 12,006 (F 9,231 / M 2,775 & (age <60 5,850 & age >=60 6,567)) treated patients with 43,846 actual test results. These were compared to the single results for 43,394 untreated patients (F 24,386 / M19,008 & Age<60 32,537 / Age>=60 10,857).

Cluster analysis showed overall for untreated patients’ median values for TSH=1.8 mUnits/L and FT4 =15.5 pmol/L, with 24% patients falling outside the 5%/95% limit, while for treated patients median TSH=3.6mUnits/L (+100% vs untreated) and FT4=18.9 pmol/L (+22%), with 22 % of treated patients falling outside the treated 5%/95% percentile boundary. When considered against the untreated boundary 75% of treated results fell outside; by sex females 78%, males 68%; by age <60 73%, >=60 74%.

**Conclusion:** The current treatment regimens being applied of either low or high dose levothyroxine are not delivering the expected laboratory TFT profiles, with significant numbers of treated patients being well outside the expected values - both TSH and FT4 being significantly higher. This effect seems to be more prevalent in women than men, which is more concerning given the higher number of women requiring treatment.

